# Cervical Spinal Cord Compression: A Rare Presentation of Hepatocellular Carcinoma

**DOI:** 10.1155/2017/8567695

**Published:** 2017-02-09

**Authors:** Puvanalingam Ayyadurai, Kanthi Rekha Badipatla, Chukwunonso Chime, Shiva Arjun, Pavithra Reddy, Masooma Niazi, Suresh Kumar Nayudu

**Affiliations:** ^1^Department of Medicine, Bronx Lebanon Hospital Center, Affiliated with Icahn School of Medicine at Mount Sinai, Bronx, NY, USA; ^2^Division of Gastroenterology, Bronx Lebanon Hospital Center, Affiliated with Icahn School of Medicine at Mount Sinai, Bronx, NY, USA; ^3^American University of Caribbean, Cupecoy, Saint Martin; ^4^Department of Pathology, Bronx Lebanon Hospital Center, Affiliated with Icahn School of Medicine at Mount Sinai, Bronx, NY, USA

## Abstract

Hepatocellular carcinoma (HCC) is the most common primary malignancy of liver. Distant metastasis to various organs is well known. Skeletal metastasis is also reported to various locations. Vertebral metastasis has been reported mostly to thoracic spine. However, cervical spinal cord involvement leading to cord compression has been reported very rarely in literature. We present a case of 58-year-old male with liver cirrhosis presenting as neck pain. Further work-up revealed metastatic HCC to cervical spinal cord resulting in acute cord compression. Patient has been treated with neurosurgical intervention.

## 1. Introduction

Hepatocellular carcinoma (HCC) is one of the aggressive neoplasms with metastatic potential [1]. Common areas of metastasis include the lung, lymph nodes, bone, and adrenal glands. Although bone metastasis of HCC has been well documented in literature with common locations being ribs, femur, pelvis, and humerus [2], metastasis of HCC to the cervical spinal cord with symptoms of spinal cord compression as the first presentation is very unusual [3–6]. We report a rare clinical scenario of a patient presenting to the hospital with neck pain ultimately leading to the diagnosis of metastatic HCC with cervical spinal cord compression.

## 2. Case

A 58-year-old male presented to the emergency room (ER) of our institution with sharp neck pain of three weeks' duration. The pain was acute in onset, radiating to right shoulder and right lateral aspect of the head. He reported that the pain was associated with numbness in right hand, blurring of vision in the right eye, tinnitus, and dizziness. Patient denied recent trauma to the head and neck. There was no history of weight loss or decreased appetite or change in bladder or bowel habits. Patient also denied headache, vomiting, chest pain, or shortness of breath.

His medical history is significant for cirrhosis of liver secondary to chronic hepatitis C infection and alcohol. His other medical conditions include essential hypertension and chronic obstructive pulmonary disease. Patient denies undergoing any prior surgical procedures. His mother was diagnosed with breast cancer. He reported smoking one pack of cigarettes for the past 40 years. He has been dependent on alcohol but denied recreational drug use.

On initial evaluation, he was afebrile and hemodynamically stable. Physical examination revealed a tender point in the posterior aspect of neck without any visible anatomical abnormalities. On neurological examination, his mental status was intact and cranial nerve examination was normal. He had increased deep tendon reflexes on right side compared to left. There was decreased sensation to touch on the right side and Romberg sign was positive. There were no signs of meningeal irritation. Cardiopulmonary and abdominal examinations were within normal limits.

His laboratory results revealed normal blood counts including platelets. His electrolytes and renal and liver function tests were within normal limits. His hepatitis C antibody was positive. He was immune to hepatitis A but not immune to hepatitis B.

**Figure 1:**
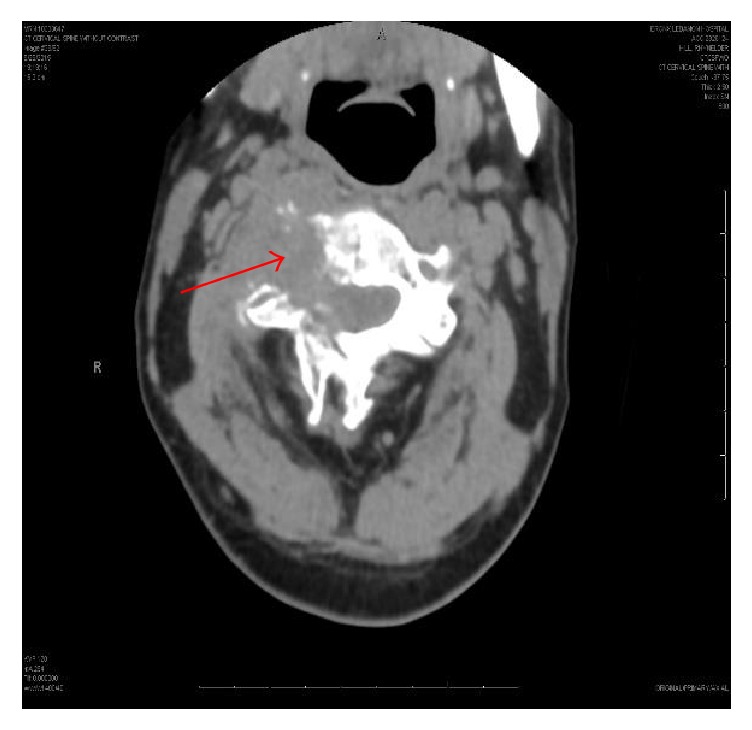
CT spine showing a mass at level of C3 with marked osseous destruction.

**Figure 2:**
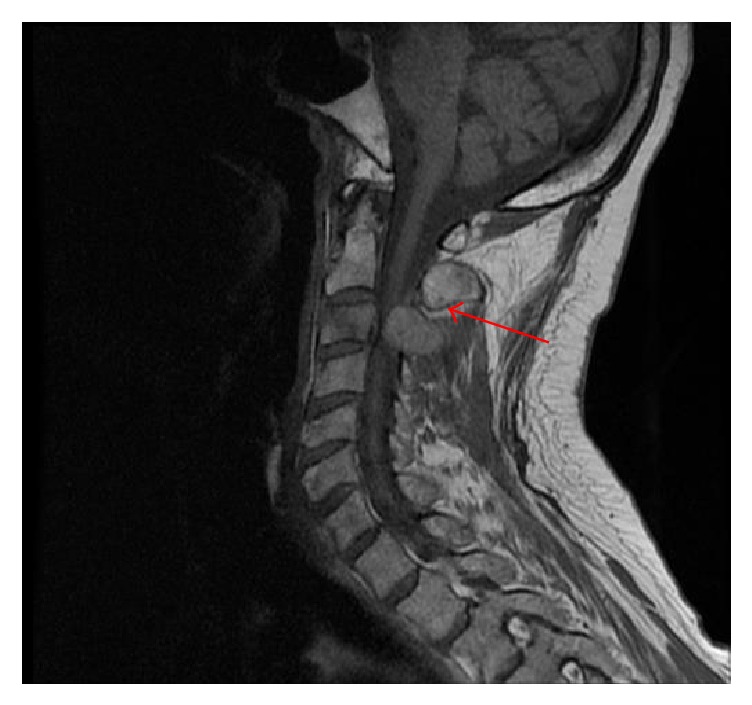
MRI of cervical spine: destructive enhancing mass at level of C2-C3 with evidence of spinal cord compression.

**Figure 3:**
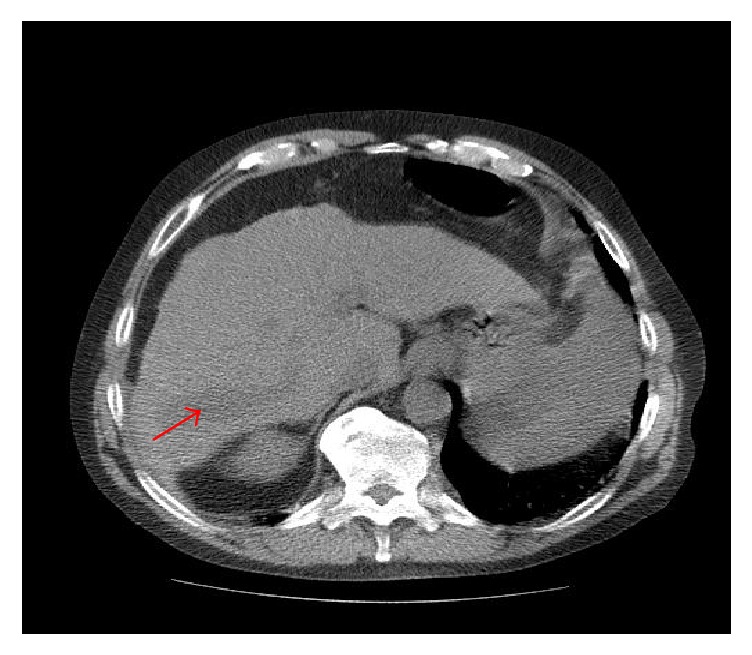
CT abdomen showing right hepatic lobe mass.

Computerized tomography (CT) of the cervical spine (Figure 1) revealed a mass at level of C3 with marked osseous destruction and encasement of right vertebral artery. Subsequent magnetic resonance imaging (MRI) of cervical spine (Figure 2) revealed destructive enhancing mass at level of C2-C3 with evidence of spinal cord compression. CT and MRI of the brain revealed no acute intracranial hemorrhage or evidence of intracranial neoplasm. CT imaging of abdomen (Figure 3) was significant for a right hemiliver mass. His laboratory results showed elevated alfa-fetoprotein (AFP) of 1167 ng/mL.

**Figure 4:**
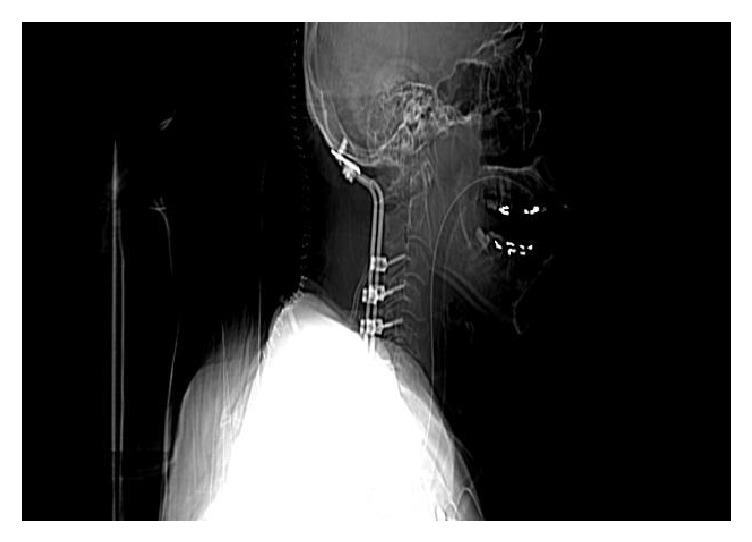
CT cervical spine after surgery showing removal of the tumor.

**Figure 5:**
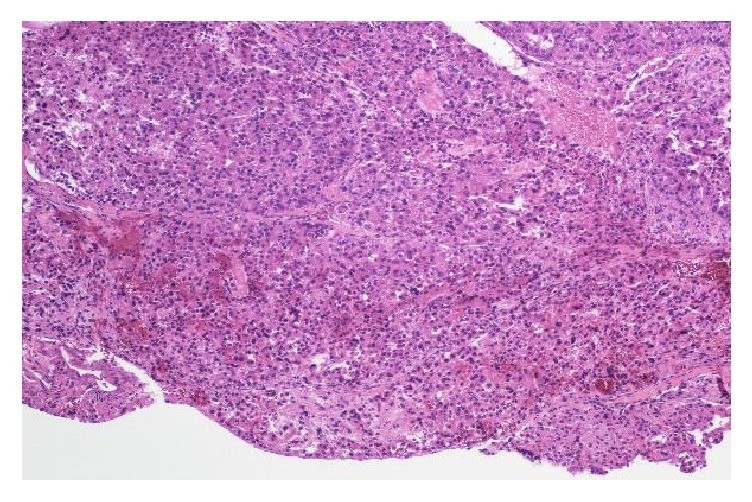
Low magnification field of the tumor showing hepatocytes with trabecular pattern of varying thickness separated by sinusoids, features consistent with HCC.

**Figure 6:**
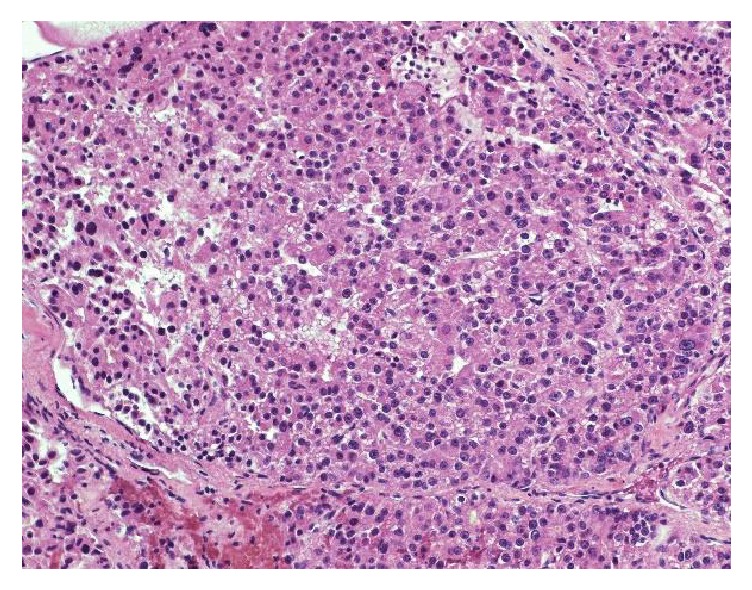
High magnification field shows thick trabecula compressed into compact mass surrounded by sinusoids.

He developed spontaneous intraperitoneal bleeding from tumor leading to drop in hematocrit. He was transferred to critical care unit for close monitoring. He was evaluated by interventional radiologist and underwent imaging guided selective arterial embolization. Subsequently upon stabilization he underwent occipital cervical stabilization and fusion (Figure 4) with excisional biopsy. Histology of the cervical mass confirmed the diagnosis of metastatic hepatocellular carcinoma (Figures 5 and 6). His hospital course was complicated by the development of pneumonia leading to septic shock and death.

## 3. Discussion

HCC is the most common primary tumor of the liver with metastatic potential [1]. Cord compression secondary to metastatic malignancy is seen with breast and lung malignancy [1]. Bone involvement in hepatocellular carcinoma is seen in about 3–20% of cases in autopsy studies [7]. Most common organs involved in HCC metastasis in the order of decreasing frequency are lung, adrenal gland and bone [8]. While bone metastasis is reported to occur in cases of hepatocellular carcinoma [2], we have noted that presentation as cervical spinal cord compression is extremely unusual.

Review of literature has revealed that so far there have been only few cases of cervical spinal cord compression due to HCC that has been reported (Table 1) [3–6, 9]. The most common presenting symptom was pain and neurological symptoms. Most of the reported cases in literature received surgical decompression while Sorafenib treatment was also discussed in literature [5].

Cord compression is an acute neurosurgical emergency and metastasis occurring in this setting will probably need surgical intervention. Further treatment of the underlying cancer itself will be based on the patient's overall general condition and severity of liver disease. Cord compression occurs due to invasion of epidural space, most often as a direct extension of vertebral body metastases. There are various routes of the epidural invasion by tumor cells, hematogenous being the most common mode of spread. Hematogenous spread occurs directly or via the involvement of Batson's venous plexuses [10].

Thoracic spinal cord appears to be the most common area of involvement. There are very few cases of HCC in literature reporting spread to the cervical spinal cord and presenting as cord compression. For most of the cases of cervical spinal cord involvement the underlying etiological hepatic risk factor appears to be hepatitis B infection. Most of the cases are reported from the east from areas of increased hepatitis B prevalence [3–5]. Nakamura et al. [9] reported four cases of cervical cord compression due to HCC in whom he evaluated the response of metastasis to radiotherapy. We are also aware in this regard that hepatitis B infection directly has the propensity to lead to hepatocellular malignancy without cirrhosis. This could probably explain the fact that there have been more numbers of reported cases associated with hepatitis B infection. Our patient is a very rare case of cervical spinal cord compression due to HCC secondary to chronic hepatitis C infection, compounded with alcohol intake.

Improvement in diagnoses and screening of HCC has led to longer overall survival of patients diagnosed with HCC. Consequently, longer survival sheds light on more extrahepatic metastases of HCC and a rise in overall metastatic HCC. Physicians should be aware of the differentials of metastatic malignancy in uncommon sites and uncommon presentations while encountering cases with underlying liver disease. Thus the awareness that cervical spinal cord involvement can occur due to HCC is of significance in initiation of early treatment. This can improve the quality of life and survival of the patients if diagnosed earlier.

## Figures and Tables

**Table 1:** Spinal cord compression due to HCC–Review of Literature.

Authors	Year	Underlying liver disease	Area of involvement	Presenting symptom	Intervention
Kantharia et al. [11]	1993	Alcoholic and hepatitis C with cirrhosis	T10	Low back pain	Steroids and palliative radiation

Yang et al. [3]	1997	Hepatitis B	Four cases with thoracic spine and one with cervical spine	Two cases with low back pain One case with hip and pelvic pain One case with abdominal pain One case with left upper extremity weakness	Palliative radiotherapy

Garcia and Castillo [12]	2005	Hepatitis B	T9	Low back pain and lower extremity weakness	High dose glucocorticoid and radiation

Doval et al. [13] (case series of 4 cases)	2006	Hepatitis B	Thoracic in 3 cases and thoracic and lumbar in 1 case	Low back pain	Chemotherapy

Nakamura et al. [9]	2006	Hepatitis B and hepatitis C	Cervical in 4 cases; thoracic spine in 15; lumbar spine in 5 cases	Neck pain and back pain	Radiotherapy

Vargas et al. [14]	2011	Hepatitis B	T4 and L5	Low back pain	Surgical decompression

Sherif et al. [15]	2012	Alcoholic cirrhosis	T12	Paraplegia	Surgical decompression

Zhang et al. [4] (case series)	2013	Hepatitis B in all cases	Five cases in cervical spine Nineteen in thoracic spine Twelve in lumbar spine	Pain and neurological symptoms	18 patients, preoperative embolization All patients, surgical decompression

Nangolo et al. [16]	2014	Chronic hepatitis B and alcohol use	L1	Lower extremity weakness	Comfort care

Vallianou et al. [6]	2014	Chronic hepatitis B	C6, C7	Upper extremity muscle pain and paresthesia	Chemotherapy and radiotherapy

Hwang et al. [5]	2015	Hepatitis B	C5 and pelvis	Upper extremity weakness and tingling	Steroid and surgery; TACE Sorafenib
